# Supplementary material for: Biomass allocation, carbon content change and carbon stock distribution of Scots pine (Pinus sylvestris var. mongholica) plantation forests at different stand ages and densities in the sandy area of western Liaoning Province, China
Source: PeerJ. 2025 Apr 4;13:e19232. doi: 10.7717/peerj.19232 (PMC11974518; doi:10.7717/peerj.19232)
Supplement: Supplemental Information 2 — The species name, age group, age and age duration in the table are derived from the National Forestry Administration ‘Classification of Major Tree Species into Age Levels and Age Groups (LY/T2908-2017)’, with YS, HMS, NMS, MS and OMS denote young, half-mature, near-mature, mature and over-mature forests, respectively. [file peerj-13-19232-s002.docx]

**1.**

**Supplement Table 1. Forest age classification criteria and age class duration of *Pinus sylvestris var. mongholica* plantation forests in northern China.**

| Tree | Age group | Age(yr) | Age-class period(yr) |
| --- | --- | --- | --- |
| *Pinus sylvestris var. mongholica* | YS | ≤20 | 10 |
|  | HMS | 21-30 |  |
|  | NMS | 31-40 |  |
|  | MS | 41-60 |  |
|  | OMS | ≥61 |  |

The species name, age group, age and age duration in the table are derived from the National Forestry Administration ‘Classification of Major Tree Species into Age Levels and Age Groups (LY/T2908-2017)’, with YS, HMS, NMS, MS and OMS denote young, half-mature, near-mature, mature and over-mature forests, respectively.

**2.**

**
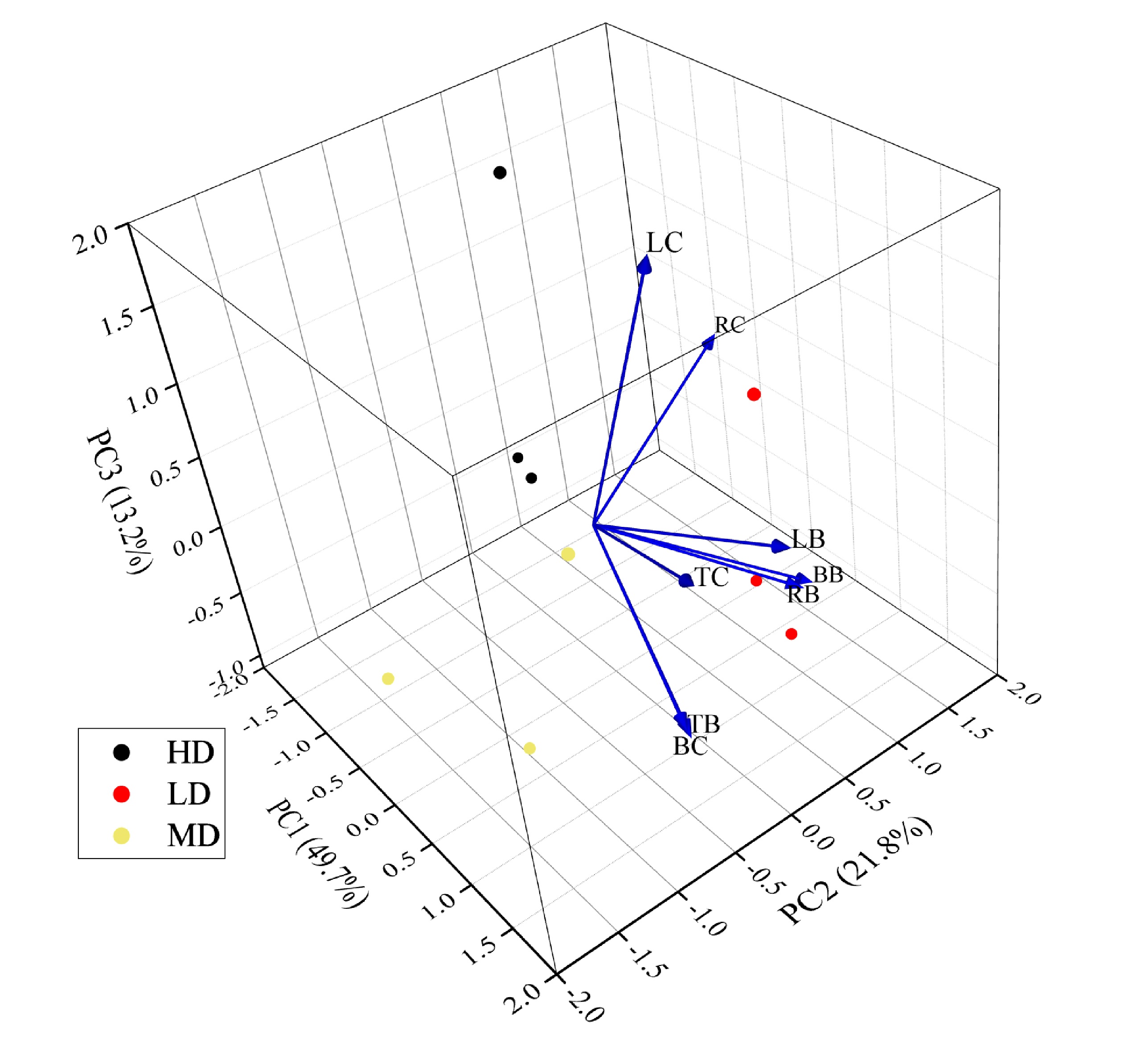
**

**Supplement Figure 1: Principal component analysis (PCA) of Scotch pine biomass and C content in different densities of near-mature forests.**

HD, MD, and LD denote high, medium, and low densities, respectively, and PC1 in the axis labels denotes the first principal component, PC2 denotes the second, and PC3 denotes the third. The percentage of principal components denotes the variance explained by the axes.

**3.**

**
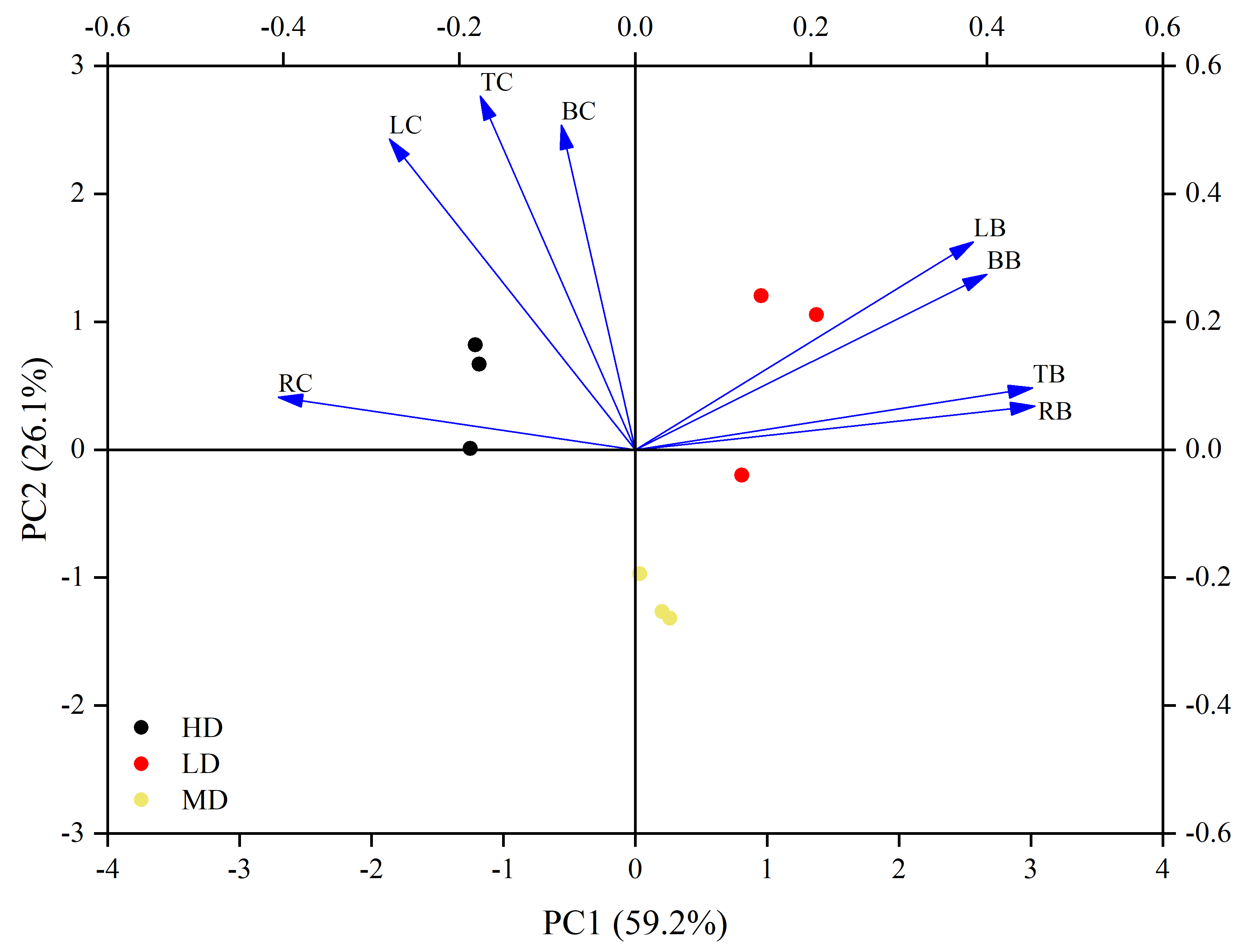
.**

**Supplement Figure 2: Principal component analysis (PCA) of biomass and C content of Scotch pine in mature forests of different densities.**

HD, MD, and LD denote high, medium, and low densities, respectively; PC1 in the axis labels denotes the first principal component, PC2 denotes the second principal component, and percentages denote the variance explained by the axes.

**4.**

**Supplement Figure 3: Distribution of carbon stock in Scotch pine in the serious land sanding area in western Liaoning Province.**

The yellow-brown color in the figure indicates severe land sanding areas and different colors indicate carbon stocks at different levels.

**5.**

The raw data of carbon stock in *Pinus sylvestris* var. *mongholica* plantation forests in western Liaoning is related to the Type II survey data of Liaoning Province, China, which is not authorised to be disclosed by the research team, please contact the Forest and Grassland Bureau of China for more details.
